# Supplementary material for: Computerized decision support is an effective approach to select memory clinic patients for amyloid-PET
Source: PLoS One. 2024 May 20;19(5):e0303111. doi: 10.1371/journal.pone.0303111 (PMC11104589; doi:10.1371/journal.pone.0303111)
Supplement: S1 Fig — AUC: appropriate use criteria, AUC+: patients fulfilling appropriate use criteria according to [13], operationalized as described in [14], PCC: probability of correct class, NP: neuropsychology, MRI: magnetic resonance imaging, Sim: simulate, FU: follow-up. (PPTX) [file pone.0303111.s001.pptx]

## Slide 1
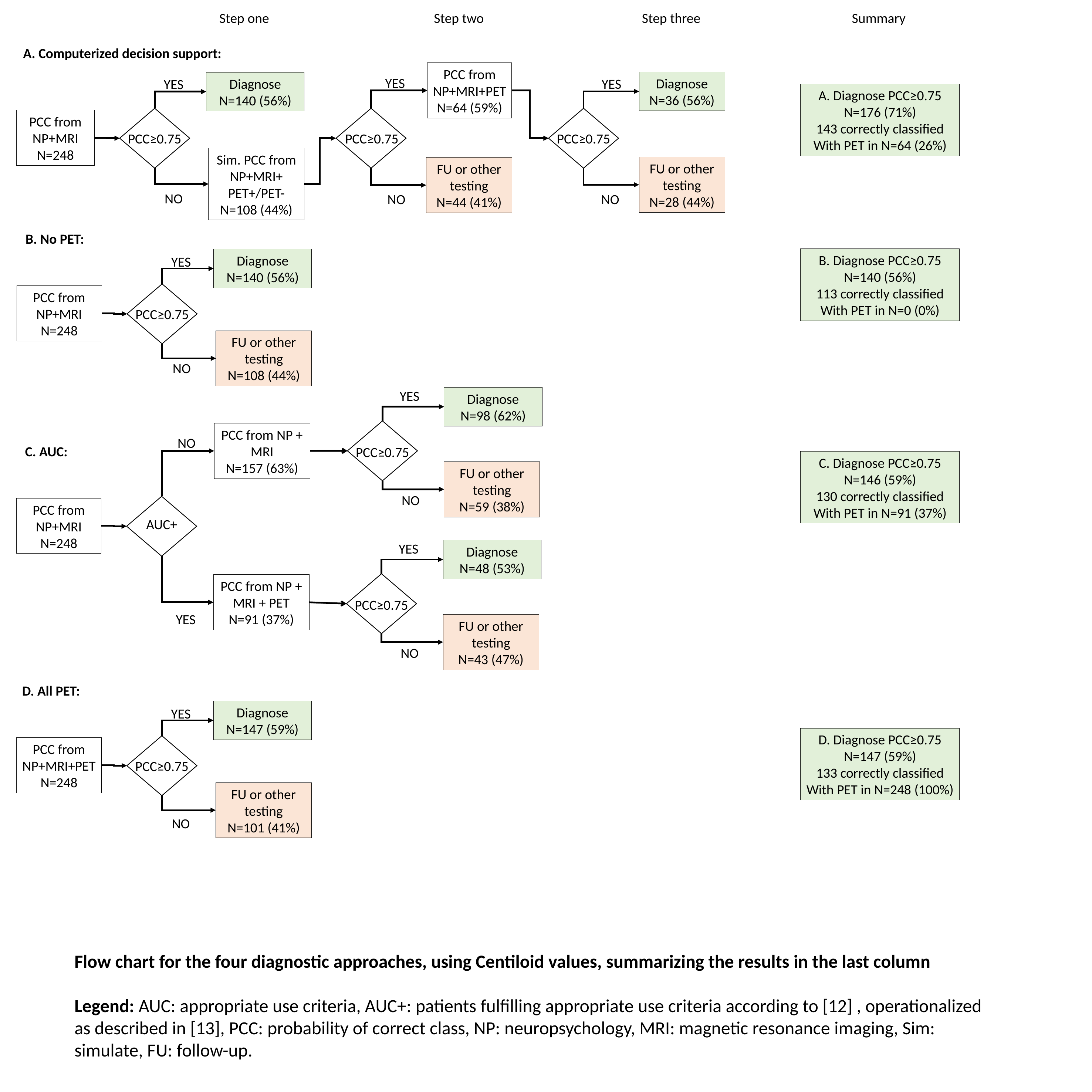

Step one
Step two
Step three
Summary
A. Computerized decision support:
PCC from
NP+MRI+PET
N=64 (59%)
YES
Diagnose
N=36 (56%)
YES
Diagnose
N=140 (56%)
YES
A. Diagnose PCC≥0.75
N=176 (71%)
143 correctly classified
With PET in N=64 (26%)
PCC from
NP+MRI
N=248
PCC≥0.75
PCC≥0.75
PCC≥0.75
Sim. PCC from NP+MRI+
PET+/PET-
N=108 (44%)
FU or other
testing
N=28 (44%)
FU or other
testing
N=44 (41%)
NO
NO
NO
 B. No PET:
B. Diagnose PCC≥0.75
N=140 (56%)
113 correctly classified
With PET in N=0 (0%)
Diagnose
N=140 (56%)
YES
PCC from
NP+MRI
N=248
PCC≥0.75
FU or other
testing
N=108 (44%)
NO
YES
Diagnose
N=98 (62%)
PCC from NP + MRI
N=157 (63%)
NO
 C. AUC:
PCC≥0.75
C. Diagnose PCC≥0.75
N=146 (59%)
130 correctly classified
With PET in N=91 (37%)
FU or other
testing
N=59 (38%)
NO
PCC from
NP+MRI
N=248
AUC+
YES
Diagnose
N=48 (53%)
PCC from NP + MRI + PET
N=91 (37%)
PCC≥0.75
YES
FU or other
testing
N=43 (47%)
NO
D. All PET:
Diagnose
N=147 (59%)
YES
D. Diagnose PCC≥0.75
N=147 (59%)
133 correctly classified
With PET in N=248 (100%)
PCC from
NP+MRI+PET
N=248
PCC≥0.75
FU or other
testing
N=101 (41%)
NO
Flow chart for the four diagnostic approaches, using Centiloid values, summarizing the results in the last column
Legend: AUC: appropriate use criteria, AUC+: patients fulfilling appropriate use criteria according to [12] , operationalized as described in [13], PCC: probability of correct class, NP: neuropsychology, MRI: magnetic resonance imaging, Sim: simulate, FU: follow-up.
