# Supplementary material for: Computerized decision support is an effective approach to select memory clinic patients for amyloid-PET
Source: PLoS One. 2024 May 20;19(5):e0303111. doi: 10.1371/journal.pone.0303111 (PMC11104589; doi:10.1371/journal.pone.0303111)
Supplement: Attachment — Submitted filename: Response to reviewers.docx [file pone.0303111.s002.docx]

*Amsterdam, January 26th 2024
Regarding PONE-D-23-25666*

*Dear dr* *Wataru Araki, Academic Editor, and esteemed Reviewers of PlosOne.*

*Please find uploaded our revised manuscript for publication in PLOS ONE ‘*Computerized decision support is an effective approach to select memory clinic patients for amyloid-PET’.

*We thank the editor and reviewers for their careful reading, thoughtful comments, and recommendation for revision. Please find below our responses to the comments in a point-by-point fashion. We have highlighted changes (via track&change) in response to the reviewers’ comments in the manuscript.*

*We hope the rebuttal adequately addresses the points raised during the review process,*

*Kind regards, also on behalf of the co-authors,*

*Hanneke Rhodius- Meester*

==================================================================================

**Journal Requirements:**

1. Please ensure that your manuscript meets PLOS ONE's style requirements, including those for file naming.
*- Reply: To our knowledge our manuscript meets the style requirements, but we are happy to make changes if needed.*

2. Please note that PLOS ONE has specific guidelines on code sharing for submissions in which author-generated code underpins the findings in the manuscript. In these cases, all author-generated code must be made available without restrictions upon publication of the work. Please review our guidelines at <https://journals.plos.org/plosone/s/materials-and-software-sharing#loc-sharing-code> and ensure that your code is shared in a way that follows best practice and facilitates reproducibility and reuse.
*- Reply: We are not sure to which code you are referring in our manuscript. Syntaxes used have been created in MatLab and have been described in previous publication (Cluitmans et al 2013* [*A MATLAB toolbox for classification and visualization of heterogenous multi-scale human data using the Disease State Fingerprint method - PubMed (nih.gov)*](https://pubmed.ncbi.nlm.nih.gov/23739361/)*), as stated in the methods section (L265-266).*

3. Thank you for stating the following in the Acknowledgments Section of your manuscript:

"Research of the Alzheimer’s Center Amsterdam is part of the neurodegeneration research program of Amsterdam Neuroscience. The Vrije Universiteit Medical Center Alzheimer Center is supported by the Stichting Alzheimer Nederland and Stichting Vrije Universiteit Medical Center Fonds. The clinical database structure was developed with funding from Stichting Dioraphte. For development of the PredictAD tool, VTT Technical Research Centre of Finland has received funding from European Union’s Seventh Framework Programme for research, technological development and demonstration under grant agreements601055 (VPH-DARE@IT) ), 224328 (PredictAD), and 611005 (PredictND). The collaboration project DAILY (project number LSHM19123-HSGF) is co-funded by the PPP Allowance made available by Health-Holland, Top Sector Life Sciences & Health, to stimulate public-private partnerships. The ABIDE clinical utility study is funded by the PPP Allowance made available by health-Holland, Top Sector Life Sciences & Health, to stimulate public-private partnerships and co-funded by Life Molecular Imaging GmbH (grant no.: LSHM18075). HR is recipient of the Memorabel Dementia Fellowship 2021 (ZonMw projectnumber 10510022110004) and Alzheimer Nederland InterACT grant (projectnumber WE.08-2022-06). LC is supported by the Innovative Medicines Initiative 2 Joint Undertaking under grant agreement No 115952. This Joint Undertaking receives the support from the European Union’s Horizon 2020 research and innovation program and EFPIA. FB is supported by the NIHR biomedical research centre at UCLH. The chair of WF is supported by the Pasman Stichting. WF is recipient of ABOARD, which is a public–private partnership receiving funding from ZonMW (number 73305095007) and Health-Holland, Top Sector Life Sciences and Health (public–private partnership allowance; number LSHM20106), WF and HR are recipient of the Horizon 2022 project PROMINENT (project number 101112145). We thank Mahnaz Shekari for her significant contribution to the processing of the amyloid-PET images."

We note that you have provided additional information within the Acknowledgements Section that is not currently declared in your Funding Statement. Please note that funding information should not appear in the Acknowledgments section or other areas of your manuscript. We will only publish funding information present in the Funding Statement section of the online submission form.

Please remove any funding-related text from the manuscript and let us know how you would like to update your Funding Statement. Currently, your Funding Statement reads as follows:

"The author(s) received no specific funding for this work."

Please include your amended statements within your cover letter; we will change the online submission form on your behalf:
*- Reply: Apologies for misunderstanding this section, we have added amended statements to our cover letter:*

***Acknowledgment Section:*** *"Research of the Alzheimer’s Center Amsterdam is part of the neurodegeneration research program of Amsterdam Neuroscience. The Vrije Universiteit Medical Center Alzheimer Center is supported by the Stichting Alzheimer Nederland and Stichting Vrije Universiteit Medical Center Fonds. The clinical database structure was developed with funding from Stichting Dioraphte. We thank Mahnaz Shekari for her significant contribution to the processing of the amyloid-PET images.*
 ***Funding Statement****: “For development of the PredictAD tool, VTT Technical Research Centre of Finland has received funding from European Union’s Seventh Framework Programme for research, technological development and demonstration under grant agreements601055 (VPH-DARE@IT) ), 224328 (PredictAD), and 611005 (PredictND). The collaboration project DAILY (project number LSHM19123-HSGF) is co-funded by the PPP Allowance made available by Health-Holland, Top Sector Life Sciences & Health, to stimulate public-private partnerships. The ABIDE clinical utility study is funded by the PPP Allowance made available by health-Holland, Top Sector Life Sciences & Health, to stimulate public-private partnerships and co-funded by Life Molecular Imaging GmbH (grant no.: LSHM18075). HR is recipient of the Memorabel Dementia Fellowship 2021 (ZonMw projectnumber 10510022110004) and Alzheimer Nederland InterACT grant (projectnumber WE.08-2022-06). LC is supported by the Innovative Medicines Initiative 2 Joint Undertaking under grant agreement No 115952. This Joint Undertaking receives the support from the European Union’s Horizon 2020 research and innovation program and EFPIA. FB is supported by the NIHR biomedical research centre at UCLH. The chair of WF is supported by the Pasman Stichting. WF is recipient of ABOARD, which is a public–private partnership receiving funding from ZonMW (number 73305095007) and Health-Holland, Top Sector Life Sciences and Health (public–private partnership allowance; number LSHM20106), WF and HR are recipient of the Horizon 2022 project PROMINENT (project number 101112145).”*

*Thank you for changing the online submission form.*

4. Thank you for stating the following in the Competing Interests section:
"We have read the journal's policy and the authors of this manuscript have the following competing interests: Hanneke FM Rhodius- Meester performs contract research for Combinostics, all funding is paid to her institution. Ingrid van Maurik received a consultancy fee (paid to the university) from Roche. Lyduine E Collij has received consultancy fees from GE Healthcare, all funding is paid to her institution. Aniek M van Gils reports no disclosures. Juha Koikkalainen and Jyrki Lötjönen report that Combinostics Oy owns the following IPR related to the paper: 1. J. Koikkalainen and J. Lotjonen. A method for inferring the state of a system, US7,840,510 B2, PCT/FI2007/050277. 2. J. Lotjonen, J. Koikkalainen and J. Mattila.State Inference in a heterogeneous system, PCT/FI2010/050545. FI20125177. Koikkalainen and Lötjönen are shareholders in Combinostics Oy. Antti Tolonen reports no disclosures. Yolande AL Pijnenburg has received funding from Dioraphte Foundation, Zabawas Foundation, JPND, ZonMW, NWO, Team Alzheimer and the Dutch Brain Foundation. Johannes Berkhof reports no disclosures. Frederik Barkhof is member of the Steering committee or Data Safety Monitoring Board member for Biogen, Merck, ATRI/ACTC and Prothena. FB is consultant for Roche, Celltrion, Rewind Therapeutics, Merck, IXICO, Jansen, Combinostics. FB has research agreements with Merck, Biogen, GE Healthcare, Roche. Co-founder and shareholder of Queen Square Analytics LTD. Elsmarieke van de Giessen has received research support from NWO, ZonMw, Hersenstichting and KWF. EvdG has performed contract research for Heuron Inc., Roche and 1st Biotherapeutics. EvdG has a consultancy agreement with IXICO for the reading of PET scans. Wiesje M van der Flier performs contract research for Biogen. Research programs of WF have been funded by ZonMW, NWO, EU-FP7, EU-JPND, Alzheimer Nederland, CardioVascular Onderzoek Nederland, Health~Holland, Topsector Life Sciences & Health, stichting Dioraphte, Gieskes-Strijbis fonds, stichting Equilibrio, Pasman stichting, stichting Alzheimer & NeuroPsychiatry Foundation, Philips, Biogen MA Inc, Novartis-NL, Life-MI, AVID, Roche BV, Fujifilm, Combinostics. WF has performed contract research for Biogen MA Inc, and Boehringer Ingelheim. WF has been an invited speaker at Boehringer Ingelheim, Biogen MA Inc, Danone, Eisai, WebMD Neurology (Medscape), Springer Healthcare. WF is consultant to Oxford Health Policy Forum CIC, Roche, and Biogen MA Inc. WF participated in advisory boards of Biogen MA Inc and Roche. All funding is paid to her institution. WF was associate editor of Alzheimer, Research & Therapy in 2020/2021. WF is associate editor at Brain"

Please confirm that this does not alter your adherence to all PLOS ONE policies on sharing data and materials, by including the following statement: "This does not alter our adherence to PLOS ONE policies on sharing data and materials.” (as detailed online in our guide for authors http://journals.plos.org/plosone/s/competing-interests). If there are restrictions on sharing of data and/or materials, please state these. Please note that we cannot proceed with consideration of your article until this information has been declared.

Please include your updated Competing Interests statement in your cover letter; we will change the online submission form on your behalf.
*- Reply: Thank you for this addition. We added an update Competing Interests statement to our cover letter, stating "This does not alter our adherence to PLOS ONE policies on sharing data and materials.”.*

5. Please include your full ethics statement in the ‘Methods’ section of your manuscript file. In your statement, please include the full name of the IRB or ethics committee who approved or waived your study, as well as whether or not you obtained informed written or verbal consent. If consent was waived for your study, please include this information in your statement as well.
*- Reply: We added the requested information on the consent procedure to the paragraph ‘Subjects’ in the Material and Methods section (L121-124).*

6. We note that you have indicated that data from this study are available upon request. PLOS only allows data to be available upon request if there are legal or ethical restrictions on sharing data publicly. For more information on unacceptable data access restrictions, please see [http://journals.plos.org/plosone/s/data-availability#loc-unacceptable-data-access-restrictions](https://eur04.safelinks.protection.outlook.com/?url=http%3A%2F%2Fjournals.plos.org%2Fplosone%2Fs%2Fdata-availability%23loc-unacceptable-data-access-restrictions&data=05%7C02%7Ch.rhodius%40amsterdamumc.nl%7Cb69d3d1f3d184680759708dbfc777996%7C68dfab1a11bb4cc6beb528d756984fb6%7C0%7C0%7C638381363374026976%7CUnknown%7CTWFpbGZsb3d8eyJWIjoiMC4wLjAwMDAiLCJQIjoiV2luMzIiLCJBTiI6Ik1haWwiLCJXVCI6Mn0%3D%7C3000%7C%7C%7C&sdata=zs59lr%2F3zU424IYhOeK9A9pxsOL9r53psPUsc%2FvUoSo%3D&reserved=0).

In your revised cover letter, please address the following prompts:

a) If there are ethical or legal restrictions on sharing a de-identified data set, please explain them in detail (e.g., data contain potentially sensitive information, data are owned by a third-party organization, etc.) and who has imposed them (e.g., an ethics committee). Please also provide contact information for a data access committee, ethics committee, or other institutional body to which data requests may be sent.

b) If there are no restrictions, please upload the minimal anonymized data set necessary to replicate your study findings as either Supporting Information files or to a stable, public repository and provide us with the relevant URLs, DOIs, or accession numbers. For a list of acceptable repositories, please see [http://journals.plos.org/plosone/s/data-availability#loc-recommended-repositories](https://eur04.safelinks.protection.outlook.com/?url=http%3A%2F%2Fjournals.plos.org%2Fplosone%2Fs%2Fdata-availability%23loc-recommended-repositories&data=05%7C02%7Ch.rhodius%40amsterdamumc.nl%7Cb69d3d1f3d184680759708dbfc777996%7C68dfab1a11bb4cc6beb528d756984fb6%7C0%7C0%7C638381363374026976%7CUnknown%7CTWFpbGZsb3d8eyJWIjoiMC4wLjAwMDAiLCJQIjoiV2luMzIiLCJBTiI6Ik1haWwiLCJXVCI6Mn0%3D%7C3000%7C%7C%7C&sdata=GMSxfV1N5%2BKLrtGbAFjFx8jDEu2syvRv8kFjKalBpRs%3D&reserved=0).

We will update your Data Availability statement on your behalf to reflect the information you provide.
*- Reply: Thank you for addressing this important point. We are afraid there are indeed restrictions to data-sharing, since the data contains potentially sensitive information (namely medical data). In the patient information folder (PIF), that subjects read before giving written informed consent, it is therefore stated specifically that their data is only shared with third parties when that data is used to gain more knowledge on Alzheimer’s disease and dementia. We can only guarantee this, via a data-request form and checking if the intended use of data aligns with the consent given by subjects. The data underlying the results presented in this study are therefore available upon request from the Data access team of the Alzheimer Center Amsterdam, via* [*wm.vdflier@amsterdamumc.nl*](mailto:wm.vdflier@amsterdamumc.nl)*. We added this statement to our cover letter.*

7. We note that you have stated that you will provide repository information for your data at acceptance. Should your manuscript be accepted for publication, we will hold it until you provide the relevant accession numbers or DOIs necessary to access your data. If you wish to make changes to your Data Availability statement, please describe these changes in your cover letter and we will update your Data Availability statement to reflect the information you provide.
*- Reply: Apologies to this misunderstanding. The data used in this study is part of the Amsterdam Dementia Cohort, of the Alzheimer Center Amsterdam, and can be retrieved from there via a data request. More information on the ADC can be found at JPND,* [*Amsterdam dementia cohort - JPND Neurodegenerative Disease Research (neurodegenerationresearch.eu)*](https://eur04.safelinks.protection.outlook.com/?url=https%3A%2F%2Fneurodegenerationresearch.eu%2Fcohort%2Famsterdam-dementia-cohort%2F&data=05%7C02%7Ch.rhodius%40amsterdamumc.nl%7C4d8681a50c6143b5295a08dbffbfab7b%7C68dfab1a11bb4cc6beb528d756984fb6%7C0%7C0%7C638384970912758466%7CUnknown%7CTWFpbGZsb3d8eyJWIjoiMC4wLjAwMDAiLCJQIjoiV2luMzIiLCJBTiI6Ik1haWwiLCJXVCI6Mn0%3D%7C3000%7C%7C%7C&sdata=dVGOD%2BF%2B3E7YkOseObn43BYh3T4ia1Fw2X42MJdy6%2FQ%3D&reserved=0)*, unfortunately without a DOI. We have updated our Data Availability statement in the cover later. Thank you for updating our statement.*

==================================================================================

**Additional Editor Comments :**
In addition to the reviewers’ comments, this editor considers that several parts in the manuscript need to be clarified or explained in more details, as follows.
1 p8 line 230-233 They cite ref13 but citing this ref. is insufficient. Please explain about AUC in more details.
- Reply: *Thank you for the opportunity to explain how we defined patients to be AUC positive or negative. De Wilde et al (ref 13) previously classified each included patient.* *In short, clinical syndrome (dementia, MCI, or SCD), suspected etiology (AD, vascular pathology, frontotemporal dementia, Lewy body dementia, other neurodegenerative disease, or non-neurodegenerative disease), and level of diagnostic confidence in suspected etiology (visual analog scale, 0–100%) were determined during pre-PET multidisciplinary meetings. After that patients were classified as AUC+ when they either i) had AD as diagnostic possibility (≥15%) but with a confidence <85% in AD as diagnosis, or ii) had a young-onset dementia (<65 years old. All other patients were classified as AUC-.This method was based on Altomare et al in 2018* [*10.1016/j.jalz.2018.02.022*](https://doi.org/10.1016/j.jalz.2018.02.022)*. We added details on this in the Material and Methods section (L243-246) and the Abstract (L34-36).*

2 Subjects in page 4 and Discussion
They included patients with AD, FTD, VaD, and SCD. Please explain why they did not include those with other dementia disorders such as DLB and AGD. In ordinary clinical practice, these non-AD dementia disorders are relatively common and should not be neglected. Clearly, this is a limitation of this study and should also be discussed.
*- Reply: Unfortunately we were not able to include other neurodegenerative diseases, such as dementia with Lewy bodies, or MCI (as the editor points out later on) since the used classifier to date does not include these patients. To reflect even better ordinary clinical practice, development of the classifier is ongoing to include more diseases as diagnostic category, such as implementing a digital cognitive test that is able to separate AD from DLB (Van Gils et al 2022* [*https://doi.org/10.1002/dad2.12379*](https://doi.org/10.1002/dad2.12379)*) and novel MRI measures to detect PSP and other Parkinson + diseases (work in progress) We added this limitation to the Discussion (L450-453, L494-497).*

3 Page 7, Ref 37 A correct ref should be described.
*- Reply: Apologies for this mistake! We have updated the reference, and refer to the PredictND study (Bruun et al 2019* [*10.2174/1567205016666190103152425*](https://doi.org/10.2174/1567205016666190103152425)*) and the Amsterdam Dementia Cohort (van der Flier et al 2018* [*10.3233/JAD-170850*](https://doi.org/10.3233/jad-170850) *(L211).*

4 Fig.2 This figure should be corrected as it does not cover the whole graphs. Fig 3 Is it possible to change the background color from blue to white?
*- Reply: We apologize for this, and have corrected both Fig 2 and Fig 3 (see Fig 2_Rhodius_revised and Fig 3_Rhodius_revised).*

5 Discussion page 14 line 403-405 “Our computerized approach even outranked performing 404 amyloid-PET in all patients” Please explain about the reasons for this result more clearly.
*- Reply: Thank you for the opportunity to elaborate on this.. Scenario A results in a higher proportion of patients diagnosed with sufficient probability as compared to the other scenario’s (including scenario D), while proportion of performed PET is much lower. This demonstrates that data-driven approaches as the one presented her might be useful in defining cost-efficient diagnostic algorithm; performing PET clearly only useful when it increases the confidence in the diagnosis. One can imagine that in borderline visual rating or Centiloid values or in case of multiple pathologies, adding PET only confuses and leads to lower proportion of diagnosed patients. We modified this sentence and added a sentence on this to the Discussion section (L405-406, L428-430 and L523).*

6. Discussion page 15 line 418-431. This editor judges that this part is not a balanced view and should be shortened or more carefully revised. It can only be said that a data-driven approach will be a supportive tool in the future clinical practice in which disease-modifying treatments become widely available. In addition, in the clinical practice, differential diagnosis of MCI patients (MCI due to AD or MCI not due to AD) is also important but this issue is not discussed in the manuscript. Why not include some discussion about this point?
*- Reply: We thank you for this suggestion to rewrite this part of discussion. We have now focused more on how this approach can help in the future, in light of DMTs, which was also a request from reviewer 1. We feel our data-driven approach can be seen as a first step to aid in coming to an accurate etiological diagnosis in an efficient way. Once an accurate diagnose is made, next step will be to define eligibility. Using the data-driven approach presented here, we will, in a new EU-project PROMINENT, further develop a stepwise approach to optimal funnel patients through the diagnostic pathway. In this approach other patients have, as the editor pointed rightly out, to be included as well, such as those with mild cognitive impairment (MCI), to aid in the relevant question if they have underlying AD or not. Novel models have to be developed to aid in this classification, of which work is ongoing. We have rewritten this part (L441-454) and hope the editor finds it now more balanced. We also added the lack of MCI in our study to the limitations (L494-497).*

7 Abstract line 48 “AUC” Please include statement about what AUC means.
*- Reply: In line with the details added on the operationalization of the AUC in the Methods section, we added a short statement on what AUC means to the Abstract (L34-36.)*

8 Abstract the last sentence “ supports clinicians in making a balanced decision in ordering additional amyloid PET during the dementia workup” This does not reflect the real situation, and should be corrected.
*- Reply: We apologize for sounding to forthcoming and have added ‘could’ to sketch possible future use (L60).*

==================================================================================

**Reviewers' comments:**

**Reviewer #1:**

With the insurance coverage of Lecanemab and other therapeutic agents against amyloid-β, there is growing interest in testing for amyloid-β detection. As the authors state in their introduction, PET scans are too expensive to be used for screening, raising the question of which patients should be aggressively screened for PET.
The purpose of this study is to "develop a computerized decision support approach to select patients for amyloid PET," but the study targets AD, FTLD, and VaD, and is more focused on disease type diagnosis rather than DMT target selection. Therefore, the study is disappointing to those who expect it to be a supportive approach to narrow down the target population for treatment of amyloid-β.
*- Reply: Indeed, our study focuses on aiding clinicians in coming to an accurate etiologically diagnosis. We apologize if this was not clear from the Introduction, and have rephrased the first paragraph to manage expectation (L67-68, 70-72, 88 and 99).
We argue however that this approach is supportive in narrowing down the target population for DMT. Due to increasing dementia prevalence and DMTs entering the market, there is a high demand for accurate etiological diagnosis, whilst keeping diagnostic work-up efficient. With the availability of DMTs, an accurate and etiological diagnosis is a prerequisite for the decision whether or not to initiate treatment. A data-driven approach will be a supportive tool in this future clinical practice, in which DMTs are widely available, to narrow done the target population for treatment. Etiological diagnosis is an important step in defining eligibility as only patient with MCI or dementia due to Alzheimer’s disease should be treated. In addition, many contraindications need to be considered. Using an adopted version of the data-driven approach presented here, we will, in a new EU-project PROMINENT, further develop a stepwise approach to optimal funnel patients through the diagnostic pathway with a specific focus on eligibility. We added this reasoning to the Discussion (L411-414, L428-430 and L441-454) and hope this is in agreement with the reviewers opinion.*

At the method of study, the following details should be considered.
1. the final classification result (likelihood of diagnosis) seems to be obtained by combining binary classification by DSI, but the rationale for "we considered that patients had a sufficiently reliable diagnosis if the PCC was 0.75 or higher" is not provided. Presumably, there is a valid basis, but it is not clear how many false positives or false negatives this may include.
*- Reply: Indeed the used the PCC cut-off of a PCC of 0.75 or higher is an arbitrary choice. In clinical practice, confidence of a clinician is closely connected to whether a clinician gives a certain diagnosis. If a clinician is not confident enough, she/he orders more tests, consults colleagues or delays diagnosis. However, this confidence is difficult to quantify as multiple factors affect it, such as expertise level, data available and how specific patterns all the tests show and impressions on meeting the patient and caregiver. PCC aims to provide a tool to define quantitatively how confident a clinician can be based on the test data. The selection of PCC cut-off is a matter of the balance between the number of diagnosed patients and the classification accuracy: the higher the cut-off is the less patients get diagnosed but with higher accuracy. As can be seen in Fig 2, the behavior is linear between cut-offs 0.6 – 0.9. Therefore, we selected a value in a middle of this range to demonstrate the algorithm in Figure 1 and Table 2. To allow clinicians freedom to set their own ‘confidence’ cut-off, the approach allows change of cut-off, as shown in Fig 2 and Fig 3. We added considerations on this to the Methods section (L194-199, L219-220) and Discussion (L458-488).*

2. it says that DSI "can handle missing data," but it is not clear how much missingness is actually allowed or how much it affects accuracy.
*- Reply: From the technical point of view, there is no limit how sparse the data can be: the classifier will perform the classification using the data that is given to it. No imputation is used.
The effect of the missing data on the classification accuracy is related to the discrimination power of the missing data. If a biomarker that is the best one to classify groups is missing, it has much higher effect on the results than if a biomarker with only a little discrimination power is missing. Therefore, it is difficult to give any cut-off, how much missing data can be tolerated. To get an idea, one of our datasets shows that MRI / MRI+MMSE / MRI+neuropsychology+CSF give 74 % / 82 % / 87 % classification accuracy for all cases and 84 % / 90 % / 96 % accuracy for the most confident 75 % cases (using a PCC cutoff) for four diagnostic groups.
As shown in Table 1, this study did not contain much missing data and therefore the impact of missing data is not highly relevant considering the results of this study. Also, the studies on how much the amount of missing data affects the classification accuracy are out of the scope of this manuscript. We hope this clarifies and answers your question.*

3. Figure 3 is helpful in understanding this study. For example, in Patient B, FTD is suspected and an amyloid PET scan is recommended as a result. However, without presentation of the present illness, symptoms, results of psychological testing, and MRI findings, the usefulness of DSI cannot be realized for readers. Wouldn't ordinary clinical diagnostic methods have been sufficient to suspect FTD?
*- Reply: Thank you for pointing our Fig 3, which is indeed meant to bridge our work to clinical practice. Indeed more clinical information is needed to value the usefulness of DSI. We therefore added clinical details to patient AD and B. We hope with this information the reviewer appreciates diagnostics was difficult in these cases. (L375-777 and L384-386).
Also, it is true that experienced clinicians can estimate diagnosis quite well even from limited information. However, dementia diagnostics is moving more and more towards quantitative biomarkers which provides an opportunity to combine all data and make diagnostics more objective and clinicians more confident. Please see our answer below on the utility of DSI for more thoughts on this.*

4. it is stated that "the strength of this study is the use of a control group and an unselected memory clinic cohort consisting of AD, FTD, and VaD patients, which reflects clinical practice," but it is not stated how PD, epilepsy, and other illness were excluded to get there.
*- Reply: Indeed, unfortunately we were not able to include other neurodegenerative diseases, since the classifier to date does not have these diagnostic groups as separate entities. We therefore only selected those patients with a diagnosis of AD, FTD, VaD and SCD in this study. Adding DLB is a natural next step, but it requires including specific tests. All other diagnoses (as recorded in our database) were not included. We added a sentence on this to the Methods section (L119).*

First of all, I would like to express my utmost respect for the results of this study. In general, the study would have been more interesting if it had been conducted with DMT in mind.
*- Reply: We agree that DMT and eligibility for DMT is highly relevant, and alters how we feel about using amyloid-PET in clinical practice. However, as argued above in response to your first comment, we feel our data-driven approach can be seen as a first step to aid in coming to an accurate etiological diagnosis in an efficient way* *as MCI and dementia due to Alzheimer’s is the key in eligibility assessment. Using an adapted version of the data-driven approach presented here, we will, in a new EU-project PROMINENT, further develop a stepwise approach to optimal funnel patients through the diagnostic pathway. We added this reasoning to the Discussion (L441-454).*

In understanding the utility of DSI, which requires the input of a variety of clinical data, what is its advantage over the diagnoses we clinicians make in our daily clinical practice? If we could understand how DSI differs from and is superior to the diagnosis that we clinicians usually make in daily clinical practice, it would be more convincing.
*- Reply: Thank you for this question on utility of DSI, which the reviewer also hinted to in the question on Fig 3. To date, clinicians have increasing diagnostics tests to use in clinical practice, aiding them in coming to a diagnosis. But we also know differential diagnosis of dementia is difficult and it takes on average 3 years for a diagnosis is made. Digital tools, like the DSI, might be useful to aid in this challenge. In a recent feasibility study in 5 Dutch memory clinics, focusing on usability, we found that clinicians valued the use of DSI because it combines all available data and presents it visually attractive for them, and also enables them to explain this to patient (we recently submitted a paper on this). In addition, we previously showed that using the DSI in clinical practice aids in clinicians confidence when setting a diagnosis (Bruun et al 2019,* [*10.2174/1567205016666190103152425*](https://doi.org/10.2174/1567205016666190103152425)*). In other words, one could consider such decision support tools as the second opinion. We hope this explains why we feel tools like the DSI can be useful in clinical practice, and added a sentence on this to the Discussion section (L476-477).*

**Reviewer #2:**

The article of Hanneke et al. affirms that a computerized decision support approach to select patients from the Memory Clinic for amyloid-PET increase the diagnostic certainty, being useful for the doctor requesting. The approach applies The Disease State Index (DSI) classifier, already tested with proven high diagnostic accuracy [references 35 and 36 of the authors] . The DSI suggests a diagnosis with a probability of correct class (PCC), based on demographics, neuropsychology, and MRI data. Thereafter, the hypothetical positive and negative amyloid-PET PCC’s values are calculated to evaluate the influence in the diagnostic certainty. The authors point out that this starting point makes the difference to other models that predict positive or negative amyloid-PET [references 40-42 of the authors]. Afterwards, an amyloid PET scan is order only if an increase to >0.75 of the PCC. Finally, the real amyloid-PET values are added to the model and PCC is evaluated.
This approach is compared with three control scenarios: without amyloid- PET using demographics, APOE, neuropsychology, and MRI; applying amyloid-PET based on The Appropriate Use Criteria (AUC) [reference 12 of the authors]; and performing amyloid-PET in all patients. Using visual PET reads and Centiloid values submit similar results to support the affirmation as a meaningful conclusion.
This proposed tool is quite relevant for the clinics, as the authors justify well in the introduction and discussion, considering nowadays there is conclusive evidence on the clinical usefulness of amyloid-PET, in the in vivo diagnosis of Alzheimer's disease (AD), as shown the results of two large international series [the American project IDEAS: reference 4 of the authors; and the European AMYPAD: reference 7 of the authors and Altomare et al. 2023]. Furthermore, even if the amyloid-PET is prescribed regarding the AUC there are other situations in which the added certainty of amyloid-PET could be helpful [reference 13 of the authors and Altomare et al. 2018]. Moreover, an increasing demand [Verger et al. 2023] is predictably because of its contribution to AD diagnosis, AD treatment (the search and introduction of possible modifying therapies [Garnier-Crussard A et al. 2023] and is the gold standard for investigating disease mechanisms [Bao et al. 2021]) and to screening improvement for clinical trials [Rabinovici et al. 2019].In words of the authors: amyloid-PET is costly and limitedly available, so intelligent and efficient use of our resources is already needed.
The statistics, with a large sample size (N=286) including controls (N=135), and other analyses (v.g. amyloid-PET procedure, Centiloid), are well conducted with a high technical standard and are described to enable reproduce. Results are exposed with sufficient detail (including mean and standard deviation) represented with clear figures and tables. The manuscript is properly presented and is written in standard English. The research meets all applicable experimentation ethics standards and integrity (blinding etc.). The article adheres to appropriate reporting guidelines and community standards for data availability.
In this work, a real-life cohort is studied (recruiting in a tertiary memory clinic) in contradistinction to the computerized decision support approach applied by the author of correspondence et al. in the cohort of Alzheimer’s Disease Neuroimaging Initiative (ADNI2) that can be consulted at Alzheimer’s Dement. 2020;16(Suppl. 5):e042687).Therefore, it cannot be considered as a replication study. The data were collected during routine care and the amyloid-PET is interpreted visually by an imaging specialist from the local hospital setting, as often do in practice and in the IDEAS project [reference 4 of the authors], and no by centralizing reading. Agreeing to the authors, this naturalistic setting features, far from being a limitation, represent an advantage due to its immediate applicability in clinical practice in the dementia workup. The strengths and limitations are well exposed.
*- Reply: thank you for this reflection. We highly appreciate your time spend on reviewing our work.*

Some aspects that may weaken the quality of the manuscript and that the authors could clarify so readers better understand could be:
DSI is not included in the abstract,
*- Reply: Thank you for pointing this out, we have added a short description of the DSI to the abstract. (L42-43).*

The data of the patients correctly classified presented in the flow chart (Figure 1) seems not be present or explained in the text.
*- Reply: Thank you for the opportunity to add this information to the text. We added the data to each scenario (L308-309, L319-320, L32 and, L 324).*

Also review: line 283 doubt about the 180 patients (66%)¿is it maybe 188 (66%)?,
*- Reply: Off course, apologies for this typo, we have corrected 180 to 188 (L308).*

line 293-297 review typographical errors and also doubt about 1654 (54%).%)¿is it maybe 154(54%)?,
*- Reply: We reviewed for errors and have corrected to the best of our knowledge, and indeed changed 1654 to 154 (L324).*

line 638: 37. !!! INVALID CITATION !!! [20, 36].
*- Reply: Apologies for this mistake! We have updated the reference, and refer to the PredictND study (Bruun et al 2019* [*10.2174/1567205016666190103152425*](https://doi.org/10.2174/1567205016666190103152425)*) {ref 37] and the Amsterdam Dementia Cohort (van der Flier et al 2018* [*10.3233/JAD-170850*](https://doi.org/10.3233/jad-170850)*. [ref 20] (L614-616, 670-672).*

Please, take as a kind suggestion the possibility of including the reference of AMYPAD 2023 in the introduction and update the reference 44.
*- Reply: Thank you for this kind suggestion, we added reference to Altomare 2023 et al in the introduction (L68, ref 8) and update ref 44 to the most recent Alzheimer facts and figures (now ref 46).*

Maybe will be interesting a brief mention about the availability of the computerized decision support approach created by the authors for use in other centers.
*- Reply: To date this method is not yet available for clinical use, the previously developed data driven approach to select the optimal patient for CSF is available via the cNeuro® tool. We added a remark on this to the discussion section (L488-490).*
