# Supplementary material for: Computerized decision support is an effective approach to select memory clinic patients for amyloid-PET
Source: PLoS One. 2024 May 20;19(5):e0303111. doi: 10.1371/journal.pone.0303111 (PMC11104589; doi:10.1371/journal.pone.0303111)
Supplement: Attachment — Submitted filename: Response to reviewers.docx [file pone.0303111.s003.docx]

*Amsterdam, April 15th 2024
Regarding PONE-D-23-25666R1*

*Dear dr* *Wataru Araki, Academic Editor, and esteemed Reviewers of PlosOne.*

*Please find uploaded our revised manuscript for publication in PLOS ONE ‘*Computerized decision support is an effective approach to select memory clinic patients for amyloid-PET’.

*We thank the editor and reviewers for their careful reading, thoughtful comments, and recommendation for re-revision. Please find below our responses to the comments in a point-by-point fashion. We have highlighted changes (via track&change) in response to the reviewers’ comments in the manuscript.*

*We hope the rebuttal adequately addresses the additional points raised during the review process,*

*Kind regards, also on behalf of the co-authors,*

*Hanneke Rhodius- Meester*

==================================================================================

**Additional Editor Comments:**

In the revised manuscript, the authors have addressed almost all the criticisms of the reviewers. However, this editor thinks that some parts are not well explained or clarified and still need to be amended as indicated below. Especially, the authors should be careful to make the manuscript more comprehensible in general.
*- Reply: We thank the editor for these suggestion and have changed the manuscript based on the comments below, and in general.*

1) Response to Editor comment 5. L405-406, L428-430? L523? These revisions are not enough to clarify the question raised by the editor.
*- Reply: The editor refers to the sentence in the original manuscript “Our computerized approach even outranked performing 404 amyloid-PET in all patients” where he during the first revision asked to explain the reasons for this result more clearly. In the revised version we rephrased this to “ Our approach was thus more efficient than the other scenario’s, where we would have performed PET in all patients, in none, or according to the appropriate use criteria (AUC).” in L405-407.*

*We are not sure what the editor seeks to clarify. The stepwise approach (scenario A) leads to 152 (53%) correctly classified subjects performing only PET in 60 (21%) subjects. Performing PET in all (scenario D) leads to 142 (49%) correctly classified subjects, yet with PET in 286 (100%) subjects. As can be seen in Fig 1, the overall accuracy is slightly higher in D, since overall less patients received a diagnosis in the approach performing PET in all. This is why we rephrased the original sentence, replacing ‘outperformed’ to ‘more efficient’. We have now rephrased this in the abstract, throughout the entire discussion and added more explanation.*

*Perhaps the editor questions why performing all tests in all is not superior to a stepwise approach? We feel our findings here are in line with several appropriate use criteria stating that an additional test should only be performed when it will increase the confidence of the clinician in a certain diagnosis. This was also added to the discussion. We hope this answer clarify the editor’s question. (L53-54, L59-60, L406, L425, L434-447, L464-465, L537, and we deleted sentence L538-539)*

2) Response to Reviewer #1 comment 4 and Editor comment 2. L119 is not enough. Please explain about the reason for this point.
*- Reply: The editor refers to his comment on “*why they did not include those with other dementia disorders such as DLB and AGD. In ordinary clinical practice, these non-AD dementia disorders are relatively common and should not be neglected”, *and the reviewer comment on* “how PD, epilepsy, and other illness were excluded to get there.”
*We totally agree that adding DLB would be useful. As explained in the methods, we were unfortunately not able to include other neurodegenerative diseases (such as DLB and AGD) or MCI, since the used classifier to date does not include these categories and therefore cannot classify them. And, though DLB is a common diagnosis in a memory clinic setting, the other diagnoses mentioned here are in fact less relevant. AGD, PD, epilepsy can sometimes occur in a memory clinic, yet are not common diagnoses (we unfortunately don’t even have AGD patients in our database) and do not pose a clinical dilemma very often. This study was set up to address the use of amyloid PET in differential diagnosis of a number of common differential diagnostic dilemma’s, in particular AD vs FTD vs VaD. We added more explanation about this to the methods section and to limitations in the discussion. We hope this explains our reason sufficiently. (L102, L118-120, L502-509)*

3) Discussion L427-429. As such, -----testing [45]. This sentence seems to be an overstatement. Please change the expression.
*-Reply: We apologize for this statement, which we added as a response to reviewer 1. We have now rephrased the sentence to ’* In addition, such approach may streamline clinical decision-making pipelines with blood-based biomarkers in the future by limiting the number of patients that require confirmatory testing [49]’. (*L465-468).*

4) Discussion L431-438 It is better to move this paragraph after the first paragraph in Discussion.
*- Reply: We thank you for this suggestion. We have now moved this paragraph after the first paragraph, and deleted 3 sentences there to prevent doubling text. (L412-425)*

5) Discussion L455-463 This paragraph is too superficial and may be omitted.
*- Reply: We have now omitted this paragraph and changed the first sentence after this paragraph to allow for better flow. (L477-485, L487)*

6) Discussion L478-500 This paragraph should be revised so that readers can understand what the limitations of this work are. Please list the limitations in order (First, second, third ----)
*- Reply: We thank the editor for this suggestion, and have rewritten the limitations sections. (L500-523)*

7) Line 482 a MCI should be MCI
*-- Reply: Apologies, we changed this into ‘*MCI*’. (L504)*

8) Line 483-485 Recruiting in ----- value. This sentence is not understandable and should be rephrased.
*- Reply: We rephrased the sentence to ‘*Second, we included only patients from a tertiary memory clinic , which may hamper generalizability, yet also reflects daily practice since amyloid-PET is mainly ordered in tertiary memory clinics’. *(L509-512)*

9)Conclusion Line 510-511 “to funnel patient through the diagnostic pathway,” This expression is not grammatically correct and should be corrected.
*- Reply: We rephrased this final sentence to ‘* to efficiently guide stepwise diagnostic testing’. *(L540-541)*

**Reviewers' comments:**

1. If the authors have adequately addressed your comments raised in a previous round of review and you feel that this manuscript is now acceptable for publication, you may indicate that here to bypass the “Comments to the Author” section, enter your conflict of interest statement in the “Confidential to Editor” section, and submit your "Accept" recommendation.

Reviewer #2: All comments have been addressed

2. Is the manuscript technically sound, and do the data support the conclusions?

Reviewer #2: Yes

3. Has the statistical analysis been performed appropriately and rigorously?

Reviewer #2: Yes

4. Have the authors made all data underlying the findings in their manuscript fully available?

Reviewer #2: Yes

5. Is the manuscript presented in an intelligible fashion and written in standard English?

Reviewer #2: Yes

6. Review Comments to the Author

Reviewer #2: (No Response)

7. PLOS authors have the option to publish the peer review history of their article (what does this mean?). If published, this will include your full peer review and any attached files.

Reviewer #2: Yes: RAQUEL SÁNCHEZ VAÑÓ

*Reply: we thank the reviewer for their efforts and are glad all comments have been addressed.*
